# Prevalence and risk factors of depression in childhood and adolescence as seen in 4 districts of north-eastern Uganda

**DOI:** 10.1186/1472-698X-13-19

**Published:** 2013-04-05

**Authors:** Eugene Kinyanda, Ruth Kizza, Catherine Abbo, Sheila Ndyanabangi, Jonathan Levin

**Affiliations:** 1 MRC/UVRI Uganda Research Unit on AIDS, Senior EDCTP Fellowship, PO Box 49, Entebbe/Cape Town, Uganda, South Africa; 2 Department of Psychiatry, Makerere University, PO Box 7072, Kampala, Uganda; 3 Mental Health Division, Ministry of Health, PO Box 7272, Kampala, Uganda

## Abstract

**Background:**

Millions of African children are having to grow up under harsh and adverse psychosocial conditions but it’s not fully understood how this negative psychosocial environment is affecting their mental health. This paper examines the prevalence and risk factors of depression in childhood and adolescence as seen in a community sample derived from four disadvantaged districts in north-eastern Uganda.

**Methods:**

1587 children were assessed using a structured instrument administered by trained psychiatric nurses to collect data on psychiatric disorders (DSM IV criteria), adverse psychosocial factors and socio-demographic factors.

**Results:**

The point prevalence of depressive disorder syndromes (DDS) in this study was 8.6% (95% CI 7.2%–10.1%) with a point prevalence for major depressive episode of 7.6% (95% CI 6.3%–9.0%) and dysthymia of 2.1% (95% CI 1.5%–3.0%). At multiple logistic regression, the factors that were independently significantly associated with DDS were: district (representing ecological factors), nature of living arrangements, domestic violence and psychiatric co-morbidities/psychiatric problems of emotional distress (assessed by the SDQ), suicidality and marginally, anxiety disorder syndromes, eating disorder syndromes, motor disorder syndromes and behavioral and developmental disorder syndromes (the later being protective against depression).

**Conclusion:**

Disadvantaged north-eastern Uganda had a high prevalence of childhood depressive disorders. Ecological factors, markers of the quality of the child-principal caregiver relationship (nature of living arrangements and domestic violence) and the presence of psychiatric co-morbidities/psychiatric problems were the important independent determinants of childhood depression in this study.

## Background

Millions of African children are having to grow up under harsh and adverse psychosocial conditions characterized by chronic war trauma, chronic poverty, HIV infection, orphan hood, child abuse and neglect, food insecurity and famine [1-4], but it’s not fully known how these conditions negatively impact on childhood mental health including how they predispose to childhood depression. A few of these adverse psychosocial conditions have been associated with childhood depression in the African situation, namely: war trauma and violence [5-10]; childhood abuse and neglect [11-13]; and orphan hood [14]. Most of the above local studies that have investigated the link between negative psychosocial factors and childhood depression have been undertaken in special population groups such as child soldiers, HIV/AIDS infected children, HIV/AIDS orphans and child laborers with a minimum of data derived from community surveys. Secondly, most of these studies considered these negative psychosocial factors singularly. Other risk factors for childhood depression mainly reported from studies undertaken in the west include: difficult family environments (characterized by marital discord, parental depression, conflict, harsh parenting style and less involved parents); stressful life events (interpersonal losses, relationship failures, divorce, bereavement and exposure to suicide); and chronic social deprivation and rejection [15,16].

Apart from two community surveys undertaken in Ethiopia which reported prevalence for childhood depression of about 1% [17,18] (similar rates of less than 2% for childhood depression have been reported in western countries [15,19]), there is a paucity of data derived from community studies on the burden of childhood depression in the sub-Saharan African socio-cultural context.

With depression projected to become the second most common cause of disability by 2020, there is need for a better understanding of its antecedents across the life span including in childhood and adolescence in different cultural settings including in sub-Saharan Africa.

This paper examines the prevalence and risk factors of depression in childhood and adolescence as seen in a community sample derived from four disadvantaged districts in north-eastern Uganda. It considers many of the identified negative psychosocial risk factors for depression simultaneously in one study exploring their relative effect on childhood and adolescent depression. It is part of a bigger project that investigated a wide range of psychiatric disorders and psychosocial problems in children and adolescents from the four districts mentioned above. A paper that investigated adolescent suicidality in this population has already been published from this work [20].

## Methods

### Study site

This study was conducted in the four districts of Lira, Tororo, Kaberamaido and Gulu in poor rural north-eastern Uganda. The study districts were selected from a list of eight districts where UNICEF was carrying out child directed medical and psychosocial interventions. In order to draw the sample of four study districts, the eight districts where UNICEF was undertaking child directed activities were subdivided into two categories of whether they were experiencing war conflict or not at the time of the study. Two study districts were then randomly selected from each of these two categories. In the category of war affected districts Gulu and Lira were selected while in the category of non-war affected districts Tororo and Kaberamaido were selected.

### Sampling procedure

Using Kish’s (1965) [21] formula for cross-sectional studies and an average district population figure based on the Uganda Housing and Population Census of 2002, a 95% confidence interval, a precision of 4% and prevalence for emotional and behavioral problems of 15% [11], a sample size for each district of 420 households was estimated. To obtain this sample from each of the study districts, a multistage sampling procedure was used. During the first stage of sampling 2 sub-counties were randomly selected from a list of all sub-counties in each of the study districts. Where the district was war affected and had part of its population living in internally displaced persons camps (IDPs), the sub-counties in that district were initially divided into two groups, those that had IDPs and those that did not have them, then from each of these two groups a sub-county was then randomly selected.

At the next stage, all the parishes in the selected sub-counties were listed and a parish randomly selected. All households in the selected parish were then listed and households with children aged 3–19 years consecutively enrolled into the study until the sample of 210 households per sub-county was attained. If the sample size of 210 household with children aged 3–19 years could not all be obtained from a single parish, a second parish would then be randomly selected from the list of parishes in that study sub-county and households could then be recruited from there until the required sample for that sub-county was obtained. Where a selected household had more than one child who was less than 19 years of age, only one study respondent was selected by simple random sampling.

### Measures

A generic survey instrument was compiled together and translated into the main dialects spoken in the selected sub-counties. To ensure accurate conveyance from English to the local dialects of the underlying psychological concepts in the items of the two main psychological assessment tools, the Strengths and Difficulties questionnaire (SDQ) [22] and the MINI International Neuropsychiatric Interview for children and adolescents (M.I.N.I.-KID) [23], a process of forward and back translation was undertaken. For each of the 4 main dialects spoken in the study sub-counties, two teams of mental health professionals were constituted. The first team translated these two psychological assessment tools into the local dialect and the second team blind to the initial English version translated the local dialect version into English. A consensus meeting with the two teams was then held and any major differences in the two versions resolved by discussions. The psychometric properties of the translated versions were not generated nor compared with each other.

The translated survey instrument were then administered by trained psychiatric nurses for each selected child aged 10–19 years or their mothers or caregiver (for those aged less than 10 years or not capable of responding verbally).

The survey instrument contained the following sections:

#### Emotional and behavioral problems

The Strengths and Difficulties questionnaire (SDQ) [22] was used to assess emotional and behavioral problems among the children and adolescents. This is a 25- item questionnaire that can be administered to parents or teachers of 3–16 year olds or directly to 11–16 year olds themselves to screen for probable psychological distress. It covers common areas of emotional and behavioral difficulties and has been validated in both western and developing country settings with good validity indices. It is scored using a Likert scale with the following scores; 0 = not true, 1 = somewhat true and 2 = certainly true. On the basis of an ROC analysis restricted to children aged 3–16 using having ‘at least one DSM IV psychiatric diagnosis’ as a ‘gold standard’, a score of at least 16 was chosen to indicate psychological distress in children. This score ensured a sensitivity of above 60% while keeping adequate specificity.

#### DSM IV psychiatric disorder

The MINI International Neuropsychiatric Interview for children and adolescents (M.I.N.I.-KID) [23] which contains DSM IV criteria for various psychiatric disorders in adolescents and children was used in this study. The psychiatric diagnoses assessed for in this study included; major depressive episode, dysthymia, manic episode, panic disorder, agoraphobia, separation anxiety disorder, social phobia, specific phobia, obsessive-compulsive disorder, post traumatic stress disorder, alcohol abuse and dependency, non-alcohol psychoactive substance use disorder, conduct disorder, oppositional deficit disorder, psychotic disorder, anorexia nervosa, bulimia nervosa, generalized anxiety disorder, adjustment disorder, pervasive development disorder, transient tic disorder, vocal tic disorder, tourette’s disorder, motor tic disorder, ADHD combined disorder, ADHD hyperactive/impulsive disorder, and ADHD inattentive disorder [23]. To construct syndrome categories that were used during analysis, the above psychiatric diagnoses were grouped as follows: depressive disorder syndromes (major depressive episode, dysthymia); psychotic disorder syndromes (manic episode, psychotic disorder); anxiety disorder syndromes (panic disorder, agoraphobia, separation anxiety disorder, social phobia, specific phobia, obsessive-compulsive disorder, post traumatic stress disorder, generalized anxiety disorder, adjustment disorder); alcohol and substance abuse disorder syndromes (alcohol abuse and dependency, non-alcohol psychoactive substance use disorder); behavioral/developmental disorder syndromes (conduct disorder, oppositional deficit disorder, pervasive development disorder, ADHD combined disorder, ADHD hyperactive/impulsive disorder, and ADHD inattentive disorder); eating disorder syndromes (anorexia nervosa, bulimia nervosa) and motor disorder syndromes (transient tic disorder, vocal tic disorder, tourette’s disorder, motor tic disorder). The psychiatric problem of life-time adolescent suicidality was defined as meeting any of the following three criteria for past suicidality as given in the MINI International Neuropsychiatric Interview for children and adolescents: i) have you ever felt so bad that you wished you were dead? ii) have you ever tried to hurt yourself? iii) have you ever tried to kill yourself? [23].

#### Socio-demographic variables

A socio-demographic questionnaire was also administered. Variables recorded include age, gender, tribe, resident district, highest level of education attained, previous history of mental illness (psychosis), attendance at a mental health facility, current living arrangement (living with both parents, mother alone, father alone, friends, adopted parents, grandparents and other relatives), orphan hood status, number of siblings, parent’s/guardian’s employment status, family’s total income per month (in Uganda shillings), parent’s highest educational attainment, nature of housing (permanent house, semi-permanent house, hut and others) and family history of mental illness (psychosis). Additional variables considered in this study included assessment for domestic violence (by asking the question: ‘in your home have you witnessed acts of domestic violence [such as threatening behaviour, violence, situations of severe beating, use of demeaning words by any of the family members?]), and for exposure to war trauma (by asking the question: ‘have you (has the child) been involved in a situation of war trauma [lived in an IDP, witnessed the torture/killing of someone, suffered physical or sexual violence as a results of war, been abducted or threatened with violence as a result of war?]).

### Ethical approval

The study obtained ethical clearance from the Ministry of Health and the Uganda National Council of Science and Technology. Respondents 18 years and above were required to provide informed consent while for children below the age of 18 years, a parents/guardian was required to consent on their behalf. Children found to have significant psychological problems were referred to the nearest government run mental health facility.

### Statistical analysis

In this study the dependent variable was the point prevalence (current disorder) of ‘depressive disorder syndromes’ (DSS) defined as having either major depressive episode or dysthymia as defined in the DSM IV derived categories in the MINI International Neuropsychiatric Interview for children and adolescents [23].

**Figure 1:**
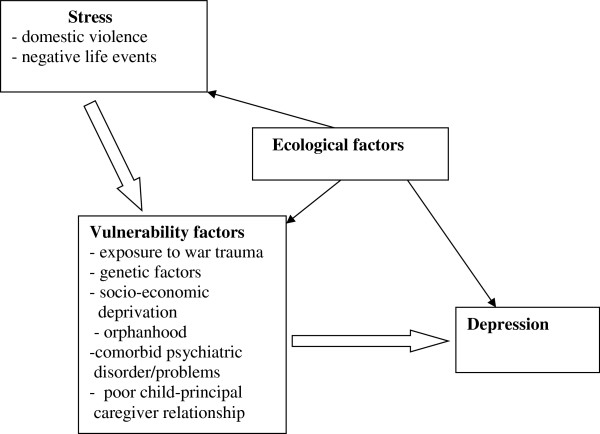
**Conceptual framework based on the stress-diathesis model for depression (Monroe & Simon, 1991) **[24]**.**

The prevalence of depressive disorder syndromes (DDS), as well as of the specific depressive disorders (major depressive episode and dysthymia), were estimated together with exact 95% binomial confidence limits, by gender and overall. A conceptual framework (Figure 1) based on the stress-vulnerability model for depression [24] was specified a priori to guide the multivariable analyses and the approach of Victoria et al. (1997) [25] was followed.

Firstly the association of socio-demographic factors was investigated through the use of a backward elimination regression model, choosing the candidate variables based on prior knowledge and plausibility, and using a liberal p-value (15%) for removal in order to ensure that all variables that could have a possible confounding effect on the ultimate risk factors were included, as recommended by Royston, Ambler and Sauerbrei (1999) [26]. The socio-demographic factors selected were then all included in a second stage model in which candidate psycho-social factors (the risk factors under consideration) were added and removed using a backward elimination algorithm using a stricter 5% nominal p-value for removal. The results were checked by carrying out forward selection with all selected socio-demographic variables included and the same candidate psycho-social factors considered. Possible differential gender effects of the identified psycho-social risk factors were investigated by testing for gender by psycho-social risk factor interactions in the final model. In addition, possible interactions between the psycho-social risk factors and the socio-demographic factors included in the final model and interactions between pairs of psycho-social risk factors were investigated. All analyses were carried out using Stata release 11.2 (Statacorp., College Station, TX).

## Results

Out of the 1680 selected respondents, the response rate for depressive disorders and other psychiatric co-morbidities was 1587 (94.5%). The main reason for non-response was repeatedly not being at home.

### Prevalence

The point prevalence (current disorder) of depressive disorder syndromes (DDS) in this study was 8.6% (95% CI 7.2%–10.1%) and was slightly higher in females (8.8%, 95% CI 7.1%–10.9%) than in males (8.3% 95% CI 6.4%–10.5%). The prevalence of DDS and that of the specific psychiatric disorders of major depressive episode and dysthymia are given in Table 1, both overall and broken down by gender. The point prevalence of major depressive episode was 7.6%, 95% CI 6.3%–9.0%), while that of dysthymia was estimated as 2.1%, 95% CI 1.5%–3.0%).

**Table 1:** Prevalence of various depressive disorder syndromes by gender

**Disorder**	**Males (n = 734)**	**Females (n = 853)**	**Total (n = 1587)**
Depressive disorder syndromes	61 (8.3%)	75 (8.8%)	136 (8.6%)
	(6.4%–10.5%)	(7.0%–10.9%)	(7.2%–10.1%)
(a) Current major depressive episode	55 (7.5%)	65 (7.6%)	120 (7.6%)
	(5.7%–9.6%)	(5.9%–9.6%)	(6.3%–9.0%)
(b) Dysthymia	11 (1.5%)	23 (2.7%)	34 (2.1%)
	(0.75%–2.7%)	(1.7%–4.0%)	(1.5%–3.0%)

### Ecological and socio-demographic characteristics of the respondents

The prevalence of DDS is summarized by socio-demographic factors in Table 2. The four districts are represented by roughly equal numbers of subjects; the prevalence of DDS depressive disorders is much higher in Lira than in the other three districts. The prevalence is higher in older subjects, but is lowest among those with some secondary education. The majority of subjects lived with both parents (945 or 59.5%) and among these participants the prevalence of DDS was lowest at 4.0%, being higher (10.7%) among those who lived with their mother only (336 or 21.2%) and highest for subjects living with their father only at 19.6% or with grandparents at 24.2%. About 8.9% of subjects (142) lived in permanent houses, and for these subjects the prevalence of DDS was much lower than for those living in huts, with the latter being the most common type of housing (1105 or 69.6%). The prevalence of DDS was highest for subjects whose guardians were unemployed (410 or 25.8%) or of “other” employment status, and lowest for those whose guardians were professional. Over a quarter of the respondents had seven or more siblings, and the prevalence of DDS was higher in this group (11.2%) than among respondents with fewer than seven siblings. The majority of respondents had parents with primary or no education (1241 or 78.2%); the prevalence of DDS was highest among respondents whose parents had no education and lowest amongst those whose parents had higher education. The socio-demographic factors chosen for inclusion in the multiple logistic regression models for DDS were district, age (grouped) and who the child lives with.

**Table 2:** Ecological and socio-demographic factors by depression in children

**Factor**	**Level**	**Total (n)**	**Depression n (%)**
District	Gulu	403	20 (5.0%)
	Kaberamaido	399	14 (3.5%)
	Lira	372	86 (23.1%)
	Tororo	413	16 (3.9%)
Age in years (grouped)	≤ 5	286	8 (2.8%)
	6–9	416	24 (5.8%)
	10–13	550	61 (11.1%)
	14–19	335	43 (12.8%)
Gender	Male	734	61 (8.3%)
	Female	853	75 (8.8%)
Education	No formal education	409	30 (7.3%)
	Primary (1–7 years)	1120	103 (9.2%)
	Secondary (8+ years)	58	3 (5.2%)
Living arrangements	With both parents	945	38 (4.0%)
	Mother Only	336	36 (10.7%)
	Father Only	56	11 (19.6%)
	With grandparents	132	32 (24.2%)
	Other	118	19 (16.1%)
Nature of housing	Permanent	133	5 (3.8%)
	Semi-permanent	297	15 (5.0%)
	Hut	1105	110 (10.0%)
	Other	52	6 (11.5%)
Family income (UGX)	< 15,000	777	90 (11.6%)
	15,000–99,000	358	25 (7.0%)
	100,000 +	285	7 (2.5%)
Employment status of guardian	Professional	338	17 (5.0%)
	Casual	382	25 (6.5%)
	Housewife	216	21 (9.7%)
	Unemployed	410	43 (10.5%)
	Other	241	30 (12.4%)
Number of siblings	0–2	243	21 (8.6%)
	3–4	421	25 (5.9%)
	5–6	493	42 (8.5%)
	7+	430	48 (11.2%)
Parent’s education	None	400	46 (11.5%)
	Elementary (Primary)	841	74 (8.8%)
	Secondary	217	12 (5.5%)
	Higher	129	4 (3.1%)

### Psychosocial and psychiatric characteristics of the respondents

Table 3 shows the prevalence of DDS broken down by psychiatric and psycho-social characteristics. The prevalence of DDS was higher among the 273 (17.1%) respondents who had experienced domestic violence (9.9% *vs* 8.3% among those who had not experienced domestic violence). DDS was more prevalent among respondents with other psychiatric disorders/problems, particularly emotional distress (as measured by an SDQ score of 16 or higher), motor disorder syndromes, psychotic disorder syndromes, eating disorders and suicidality, and for the 86 (5.4%) who had two or more co-morbidities the prevalence of DDS was 44.2%. The prevalence of DDS was higher among those who had experienced war trauma than among those who had not experienced war trauma (10.8% *vs.* 7.3%). It was higher among subjects whose parents were not both alive. Surprisingly it was lower among subjects with a history of mental illness.

**Table 3:** Psychiatric disorder/problems and psychosocial factors by depression in children

**Factor**	**Level**	**Total (n)**	**Depression n (%)**
**Psychosocial Factors**			
Domestic violence	No	1314	109 (8.3%)
	Yes	273	27 (9.9%)
Family history of mental illness	None	1175	104 (8.8%)
	First Degree relative	156	15 (9.6%)
	Other relative	256	17 (6.6%)
Experience of war trauma	No	1023	75 (7.3%)
	Yes	564	61 (10.8%)
Parents alive	Yes	1069	47 (4.4%)
	No	518	89 (17.2%)
History of mental illness (attendance at facility)	No	1517	132 (8.7%)
	Yes	70	4 (5.7%)
**Psychiatric disorder/Problems**			
Anxiety disorder syndrome	No	1164	59 (5.1%)
	Yes	423	77 (18.2%)
Psychotic disorder syndromes	No	1563	126 (8.1%)
	Yes	24	10 (41.7%)
Suicidality	No	1502	81 (5.4%)
	Yes	85	55 (64.7%)
Alcohol and substance abuse disorders	No	1565	130 (8.3%)
	Yes	22	6 (27.3%)
Motor disorder syndromes	No	1574	129 (8.2%)
	Yes	13	7 (53.8%)
Behavioral & developmental disorder syndromes	No	1508	128 (8.5%)
	Yes	79	8 (10.1%)
Eating disorders	No	1576	130 (8.2%)
	Yes	11	6 (54.6%)
Number of DSM disorders	0	1056	41 (3.9%)
	1	445	57 (12.8%)
	2 or more	86	38 (44.2%)
Emotional distress (assessed by SDQ scores)	Non-case (SDQ < 16)	918	45 (4.9%)
	Case (SDQ ≥ 16)	669	91 (13.6%)

### Multivariable associations of childhood depression

The results of fitting multiple logistic regression models including the socio-demographic factors identified as being important is given in Table 4. Adjusting for district, age and living arrangements, the factors found to be significantly associated with DDS were experience of domestic violence, with subjects who experienced domestic violence being more likely to have DDS (aOR 1.94, 95% CI 1.05–3.56, P = 0.038). There is also overwhelming evidence that subjects with psychiatric disorders/problems were more likely to have DDS, particularly subjects who exhibited suicidality (aOR 27.62, 95% CI 13.86–55.06) and emotional distress (as shown by an SDQ score of 16 or higher (aOR 3.30, 95% CI 1.83–5.01). Marginally associated with DDS were the psychiatric disorder syndromes of eating disorder syndromes, anxiety disorder syndromes and motor disorder syndromes. Surprisingly participants with behavioral or developmental syndromes were significantly less likely to have DDS (aOR = 0.24; 95% CI 0.06–1.00). Using the conceptual framework approach, the effects of the socio-demographic variables should be interpreted as those effects not mediated through the psycho-social variables Victoria et al. (1997) [25]. Two factors have such effects, notably district (respondents from Lira were most likely to have DDS whilst those from Kaberamaido were least likely to have DDS) and participants living with both parents are least likely to have DDS; compared to these, all other participants were significantly more likely to have DDS, particularly those living with grandparents or non-relatives. Interactions between gender and psycho-social risk factors, between socio-demographic factors in the final model and psycho-social risk factors and between pairs of psycho-social risk factors were examined; no interaction was found to be significant at the 5% level. Note that at both model selection stages (choosing the socio-demographic factors and choosing the psycho-social factors) forward selection confirmed the factors chosen by backward elimination, reflecting the fact that relatively few candidate variables were considered based on prior knowledge.

**Table 4:** Results of fitting multiple logistic regression models for factors associated with depression in children

**Factor**	**Level**	**Odds ratio (95% Confidence interval)**	**Likelihood ratio test P-value**
District	Gulu	1 (Reference level)	<0.0001*
	Kaberamaido	0.19 (0.08; 0.47)	
	Lira	3.84 (2.06; 7.16)	
	Tororo	0.44 (0.18; 1.06)	
Age group	≤ 5	1 (Reference level)	0.26
	6–9	1.44 (0.55; 3.74)	
	10–13	2.09 (0.87; 5.01)	
	14–19	1.48 (0.57; 3.84)	
Living arrangements	Both Parents	1 (Reference level)	<0.0001*
	Mother Only	2.18 (1.20; 3.96)	
	Father Only	3.08 (1.14; 8.33)	
	With Grandparents	6.21 (3.17; 12.15)	
	Other	5.36 (2.51; 11.43)	
Experience of domestic violence	No	1 (Reference level)	0.038*
	Yes	1.94 (1.05; 3.56)	
Emotional distress (assessed by SDQ scores)	Non case (SDQ < 16)	1 (Reference level)	<0.0001*
	Case (SDQ ≥ 16)	3.03 (1.83; 5.01)	
Motor disorder syndromes	No	1 (Reference level)	0.075
	Yes	10.72 (1.00; 114.6)	
Eating disorders	No	1 (Reference level)	0.049*
	Yes	12.94 (1.25; 133.4)	
Behavioral disorders	No	1 (Reference level)	0.027*
	Yes	0.24 (0.06; 1.00)	
Anxiety	No	1 (Reference level)	0.047*
	yes	1.67 (1.01; 2.76)	
Suicidality	No	1 (Reference level)	<0.0001*
	Yes	27.62 (13.86; 55.06)	

Note: * Statistically significant association.

## Discussion and conclusion

This is one of a few African studies in the published literature that has examined the prevalence and risk factors of childhood depressive disorders in a community sample. The principal finding of this study is that north-eastern Uganda had a high point prevalence of childhood depressive disorders. Secondly, that the ecological factors (represented by district in this study), markers of the quality of the child-principal caregiver relationship (nature of living arrangements and domestic violence) and the presence of psychiatric co-morbidities were the important independent determinants of childhood depression in this study.

This study was carried out in four disadvantaged districts of north-eastern Uganda two of which were suffering war conflict at the time of the study. Most of respondents were children of poor peasant farmers with little formal education and largely operating at subsistence level outside the formal cash economy. These study communities had a heavy burden of adverse psychosocial factors, domestic violence was reported by a fifth of the respondents, war trauma was reported by a third of the respondents, orphan hood was reported by a third of the children and only half of the children stayed with both parents.

The point prevalence of childhood depressive disorder syndromes (DDS) of 8.6% in this study is much higher than the rate of about 1% that has been reported in both rural and urban Ethiopia [12,13] and of less than 2% reported in western studies [15,19]. The prevalence of depression in this study (where half of the study districts were war affected) is however lower than that of 19.6% reported in war affected Sri Lanka [27]. Attanayake and colleagues (2009) [8] in a meta-analysis involving 17 studies on mental disorders among children exposed to war reported a 43% elevation in the risk of depression associated with exposure to war. The results from the above mentioned meta-analysis suggest that the elevated prevalence of childhood depression observed in this study relative to the results from Ethiopia may have been due to the fact that half of the study districts were suffering ongoing war conflict at the time of the research.

Ecological, socio-demographic, adverse psychosocial and psychiatric factors were each independently significantly associated with childhood depression in this study. District in this study representing ecological factors was independently significantly associated with depression with the non-war affected districts of Kaberamaido (3.5%) and Tororo (3.9%) reporting lower rates of depression than the war affected districts of Gulu (5.0%) and Lira (23.1%). However the marked difference between the prevalence of depression in Gulu and Lira which were both war exposed suggests that there were additional area level factors at play influencing the district prevalence for depression. In this study it was not possible to investigate further the factors underlying these area level differences in the rates of depression. Among adults, Vinck and colleagues (2007) [28] in 4 war affected districts in north-eastern Uganda and Kinyanda and colleagues (2009) [29] in a 14 district study in Uganda observed similarly wide variations in district rates of depression. Among the investigated factors to account for these area level differences, Kinyanda and colleagues (2009) reported that literacy rates were inversely significantly associated with the district rates of depression [29].

In this study, the socio-demographic factor, ‘nature of living arrangement’ was independently significantly associated with depression. Children not living with both parents had an increased risk of developing depression which was higher in those staying with a single parent (either mother or father) and highest in those staying with neither parent (staying with grandparents or with non-relatives). The fact that in this study domestic violence was also independently significantly associated with childhood depression but not orphan hood seems to suggest that the important underlying construct was the quality of the ‘child-principal caregiver relationship’ but not just the absence of a biological parent(s). Indeed emotional unavailability of both mothers and fathers has consistently been reported to be a risk factor of childhood depression [30,31]. In central Uganda, Minde (1975) [11] observed that polygamous family set-ups were associated with psychological problems in school going children. Sander and McCarty (2005) on parental risk factors for depression reported that these included parents who were: emotionally unavailable or uninvolved, who lacked warmth in their interactions, are overcontrolling, or who use harsh physical discipline [30]. On domestic violence, Nicodimous and colleagues (2009) [12] among college going adolescents in Ethiopia reported that witnessing parental violence was associated with more than a 2 fold increased risk for depression.

In this study, co-morbidity with the psychiatric disorders/problems of emotional distress (assessed by the SDQ scale), suicidality, eating disorders and marginally anxiety disorder syndromes were independently significantly associated with childhood depression. Co-morbidity between depression and behavioral and developmental disorder syndromes was significantly protective against depression in this study. Pataki (2000) [15] on the epidemiology of childhood and adolescent depression observes that between 40–70% of children and adolescents with depression will have a co-morbid psychiatric disorder usually anxiety disorder, dysthmic disorder, disruptive behavioral disorder or a substance abuse disorder. In this study, surprisingly the rate of DDS was lower among subjects with a history of mental illness. Two plausible explanations can be offered for this observation. Firstly, given that only four participants had a history of mental illness, the small sample size may have led to spurious results. Secondly, professional help provided during attendance at the mental health facility may have effectively resolved the psychological problem hence lower levels of DDS.

In this study we found no significant independent effect for measures of socio-economic deprivation (nature of housing, family income, highest educational attainment of parent/guardian) and some of the adverse psychological factors (experience of war trauma and orphan hood). It is possible that these could be risk factors for childhood depression, acting indirectly through affecting the quality of the child-principal care-giver relationship or leading to psychiatric comorbidities.

In conclusion, there is a high prevalence of childhood major depressive disorders in disadvantaged north-eastern Uganda. Ecological factors, quality of the child-principal care-giver relationship and the presence of psychiatric co-morbidities were the important independent determinants of childhood depression in this study. We found that socio-economic deprivation, experience of war trauma and orphan hood were not significantly associated with depression, but it is possible that these factors had an indirect effect on depression through affecting either the child-principal care-giver relationship or leading to associated psychiatric co-morbidities.

On limitations, firstly, given that this study was cross-sectional in nature the causal direction between the dependent and the independent factors could not be ascertained, hence there is need for longitudinal studies to disentangle the casual directions between these factors. Secondly, the psychological assessment tools used in this study have never been formally validated in the Ugandan culture settings, however the SDQ has previously been validated in Congo-DRC (a socio-cultural setting similar to Uganda) with good results [32]. In addition, to ensure accurate translation of the SDQ and the M.I.N.I.-KID from English into the 4 local dialects used in this study, a process of forward and back translation was undertaken using teams of local mental health professionals. Thirdly, because 4 dialect versions of the questionnaire were used in this study, reported district differences could be due to bias introduced by the translation process, this effect was however thought to be minimal as the process of forward and backward translation was undertaken carefully. Fourthly, this paper presents the results of a secondary analysis of data, and important data relating to the sample selection was not available, in particular it would have been helpful to have data on the age and sex composition of children in the sampled households, including children who were not included in the sample, in order to ascertain whether the age and sex distributions of sampled children were representative of children in each district. Korn and Graubard (1995) suggest that weighting the data to reflect the underlying age and sex distribution of the districts would impact on the estimated prevalence of depressive disorders but not on the estimates of association for the risk factors [33].

As recommendations, addressing childhood depressive disorder in this community requires both social interventions to improve the quality of the child-principal caregiver relationship (through interventions such as training in parenting skills and domestic violence counseling) and the development of comprehensive child and adolescent mental health services (to address the entire spectrum of childhood psychopathology due to a tendency for co-morbidity). There is a need for the formal validation of the SDQ and M.I.N.I.-KID in the African socio-cultural setting of Uganda.

## Abbreviations

EK: Eugene Kinyanda; RK: Ruth Kizza; CA: Catherine Abbo; SN: Sheila Ndyanabangi; JL: Jonathan Levin.

## Competing interests

The authors declare that they have no competing interests.

## Authors’ contributions

Concept: RK, CA, SN, EK; Data collection: RK, CA, SN, EK; Data analysis: EK, JL; First draft: EK, RK, CA, SN, JL; Final revision: EK, RK, CA, SN, JL. All authors read and approved the final manuscript.

## Pre-publication history

The pre-publication history for this paper can be accessed here:

http://www.biomedcentral.com/1472-698X/13/19/prepub
